# Clinical characteristics and outcomes of chronic nonbacterial osteomyelitis in children: a multicenter case series

**DOI:** 10.1186/s12969-021-00657-4

**Published:** 2022-01-03

**Authors:** Le Ma, Haimei Liu, Hanyun Tang, Zhiyong Zhang, Lixia Zou, Haiguo Yu, Li Sun, Xiaozhong Li, Xuemei Tang, Meiping Lu

**Affiliations:** 1 Department of Rheumatology and Immunology, Children’s Hospital of Nanjing Medical University, Nanjing, China; 2 Department of rheumatology, Children’s Hospital of Fudan University, Shanghai, China; 3 Department of Nephrology and Immunology, Children’s Hospital of Soochow University, Suzhou, China; 4 Department of Rheumatology and Immunology, Children’s Hospital of Chongqing Medical University, Chongqing, China; 5 Department of Allergy Immunology and Rheumatology, Children’s Hospital of Zhejiang University School of Medicine, Hangzhou, China

**Keywords:** Autoinflammatory bone disease, Chronic nonbacterial osteomyelitis, Chronic recurrent multifocal osteomyelitis

## Abstract

**Objective:**

The aim of this study was to evaluate demographic, clinical, laboratory, imaging, histopathology characteristics, and treatment responses of children with Chronic nonbacterial osteomyelitis (CNO).

**Methods:**

Retrospective multi-center case series study of pediatric patients diagnosed with CNO treated at five tertiary centers in south China.

**Results:**

Totally there were 18 patients diagnosed as CNO between 2014 and 2020. The median age of onset was 9.2 years (range 3.7–13.1) and 55.6% were female. Median delay in diagnosis was 10.9 months (range 1.0–72.0). The most frequent presenting symptoms were bone pain (100%) and fever (44.4%). Most patients had more than one lesion (median of 5, range 1–7). Most frequently affected bones were tibiofibula (88.9%) and femur (77.8%). The MRI characteristics mainly presented as bone edema and hyperintensity in bone marrow. Bone biopsy was conducted in 11 patients (61.1%) with inflammatory cells infiltration manifested as chronic osteomyelitis, and none showed bacterial infection or tumor. In treatment, non-steroid anti-inflamatory drugs (NSAIDs) is used as the first-line drug followed by steriods, methotexate (MTX), salazosulfadimidine (SASP), Bisphosphonates and TNF-α inhibitor. Two refractory cases received combination therapy with Bisphosphonates and TNF-α inhibitor, and achieved good therapeutic effect.

**Conclusions:**

The present study described a multicenter series of CNO from south China and highlighted the clinical features, laboratory tests, imaging characteristics and treatment outcomes. Increasing awareness of this disease is important to decrease time to diagnosis, improve access to treatment, and reduce complications.

## Introduction

Chronic nonbacterial osteomyelitis (CNO) is a benign and noninfectious autoinflammatory bone disease that mostly affects children and adolescents [1–3]. In 1972, Giedion et al. first described four patients with subacute or chronic multifocal symmetrical osteomyelitis, which mostly affects the growth plates of long bones [4]. Some scholars subsequently described similar cases and gave the diagnostic term “chronic recurrent multifocal osteomyelitis (CRMO)” [5]. Recurrent flares of inflammatory bone pain related to aseptic osteomyelitis are the major symptoms of the disease. The clinical presentation varies widely, from mild, unifocal, and time-limited bone involvement to severe, chronically active, or recurrent disease with multifocal bone lesions [5–8]. Thus, the name “CNO” has been proposed to encompass them all [9]. In adults, the term SAPHO syndrome is commonly used when referring to manifestations of the disease, which include synovitis, acne, pustulosis, hyperostosis, and osteitis [10]. To date, whether CNO and SAPHO syndrome are different manifestations of the same disease at different ages of onset or different outcomes of different clinical manifestations of the same disease remains unresolved [11].

Epidemiological data on CNO from Chinese population are limited. The average age at onset of the disease is 7–12 years, but the delay in diagnosis from the onset of symptoms is usually around 1 year [12]. A study conducted in Germany reported an incidence of 0.4 per 100,000 children [13]. The current incidence of the disease in other regions is unknown. The true incidence of CNO has likely been underestimated in past studies [14]. In most multicenter studies from North America and Europe, there is a female predominance with a female:male ratio of approximately 2:1. However, in similar studies from India and Japan, a male predominance is observed [8, 12]. To date, CNO is usually diagnosed by exclusion with magnetic resonance imaging (MRI) and/or through bone biopsy, which reveals chronic inflammation without infectious and oncological agents. The treatment protocol for CNO includes NSAIDs, steroids, MTX, SASP, TNF-α inhibitors, and bisphosphonates.

Although the awareness of CNO has increased over the past decade, misdiagnosis and delays in treatment still persist. Reliable diagnostic criteria and treatment protocols are lacking, and more multicenter studies on CNO are needed. To improve our understanding of the disease, we sought to evaluate the demographic and clinical characteristics, radiological findings, as well as treatment responses in pediatric patients with CNO at five tertiary centers in South China.

## Methods

Patients under 18 years old diagnosed with CNO between 2014 and 2021 were enrolled at five tertiary centers in South China: Children’s Hospital of Nanjing Medical University, Children’s Hospital of Fudan University, Children’s Hospital of Soochow University, Children’s Hospital of Zhejiang University School of Medicine, and Children’s Hospital of Chongqing Medical University. The study was approved by local Ethics Review Board, and granted exemption of informed consent. Clinical characteristics, such as age, sex, family history, age at onset of symptoms, delay in diagnosis, symptoms, comorbidities, and treatments, were recorded. Available laboratory evaluation images, including plain radiographs, computed tomography (CT), Tc99 bone scan, MRI, and histology from bone biopsy were collected. Literature review was conducted on the case series of CNO reported abroad, and clinical comparison was made among our study and foreign CNO cohorts.

The diagnosis of CNO was defined as the presence of unifocal or multifocal inflammatory bone lesions with radiological and/or histopathological characteristics compatible with this diagnosis [15]. Infectious, oncological, or other inflammatory diseases were excluded. Response to treatment was assessed by improvement in pain and serologic markers of inflammation as well as radiographic proof of bone healing. The pain was evaluated by Visual Analogue Scale/Score. “No response” was defined as persistent pain with elevated inflammatory markers and abnormal signal on MRI.“Complete response/Remission” was defined as an 80% or more remission of clinical symptoms and imaging manifestation as well as normal inflammatory markers. The remission of 20% ~ 80% is “partial response”.

### Statistical analyses

All statistical analyses were performed using SPSS 17.0. Continuous variables with normal distribution were presented as mean ± SD or median (IQR or range).

## Results

### General

Between 2014 and 2021, 18 patients were enrolled. Main demographic and clinical characteristics are described in Table 1. The median age at onset of the disease was 9.2 years (range 3.66–13.08) and 55.6% (*n* = 10) were female. The median time from onset to diagnosis was 10.9 months with a range of 1–72 months. The median follow-up was 16 months.

**Table 1:** Characteristics of patients with chronic nonbacterial osteomyelitis

Characteristics	Our patients
Demographics	
Age at disease onset, years, median (range)	9.2 (3.66–13.08)
Female, n (%)	10 (55.6%)
Delay in diagnosis, months, median (range)	10.9 (1–72)
Follow-up, months, median (range)	16 (3–54)
Clinical features	
Distribution of involvement	
Skull, *n* (%)	0
Nasal bone, *n* (%)	0
Cheekbone, *n* (%)	0
Mandible, *n* (%)	1 (5.6%)
Clavicle, *n* (%)	2 (11.1%)
Sternum, *n* (%)	1 (5.6%)
Ribs, *n* (%)	1 (5.6%)
Humerus, *n* (%)	4 (22.2%)
Radius and ulna, *n* (%)	6 (33.3%)
phalanges**,*n*(%)**	2 (11.1%)
Spine, *n* (%)	3 (16.7%)
Pelvis, *n* (%)	3 (16.7%)
Femur, *n* (%)	14 (77.8%)
tibiofibula, *n* (%)	16 (88.9%)
calcaneus**,*n*(%)**	4 (22.2%)
Initial symptoms, *n* (%)	
Bone pain	18 (100%)
Limp	5 (27.8%)
Swelling	4 (22.2%)
Fever	8 (44.4%)
Comorbidities	
arthritis, *n* (%)	8 (44.4%)
Uveitis, *n* (%)	0
gastrointestinal symptoms	1 (5.6%)
Palmoplantar pustulosis	0
psoriasis	0
acne	1 (5.6%)
NBO score, median (range)	39 (30–53)
CNO Family history	0
HLA-B27 positivity, %	0
ANA positivity, %	0
ESR at initial visit (mm/h)	57.5 ± 42.2
ESR elevated,%	12 (66.7%)
CRP at initial visit (mg/L)	48.8 ± 48.8
CRP elevated,%	11 (61.1%)

*CRP* C reactive protein, *ESR* erythrocyte sedimentation rate

### Clinical presentation

Most of the patients had a recurrent multifocal disease pattern, and a median of 5 bone lesions (range 1–7). Bone lesions affecting the appendicular skeleton were seen in 100%, including 38.9% (*n* = 7) in the upper limbs and 88.9% (*n* = 16) in lower extremities; while 38.9% (*n* = 7) had axial skeleton involvement. Most frequently affected bones were femur (77.8%), tibiofibula (88.9%), radius and ulna (33.3%), humerus (22.2%) and calcaneus (22.2%). Two patients had clavicle involvement. In one of these patients, CNO only affected the left clavicle, while in another one also affected mandible, sternum and lower extremity bones. One patient (case 18) had Right 12th rib and cervical spine involvement.

The initial symptom was bone pain in all patients. 22.2% presented local swelling, 27.8% limp, and 44.4% fever. Of the 18 patients, 8 (44.4%) had comorbid inflammatory arthritis. Case 18, who had rib and cervical spine involvement, also had mild acne, enthesitis and a appendix ulcer, which didn’t meet the diagnostic criteria of inflammatory bowel disease or Behcet’s disease. No patients in this series had psoriasis, inflammatory bowel disease, palmoplantar pustulosis, uveitis or severe acne. No patients had a first- or second-degree relative with history of autoimmunity.

The clinical scores for Nonbacterial Osteitis (NBO scores) was used to help diagnosis, which provided by Annette F. Jansson et al. [16]. The average score of our patients was 39 points, ranged from 30 points to 53 points.

### Laboratory tests

In terms of laboratory studies, normal white blood counts were observed at onset in most patients, except for two patients with mild leukocytosis. Mean erythrocyte sedimentation rate (ESR) was 57.5 ± 42.2 mm/h, and 12 patients (66.7%) had elevated ESR over 20 mm/h. Mean C-reactive protein (CRP) was 48.8 ± 48.8 mg/L, 11 patients (61.1%) had CRP over the normal value of 8 mg/L. In 9 patients, both CRP and ESR were elevated simultaneously. No patients had positive ANA or positive HLA-B27. In all cases, bacterial cultures were negative. Four patients accepted genetic testing. Except for several known polymorphisms, no mutations were detected.

### Imaging findings and bone biopsy

**Fig. 1:**
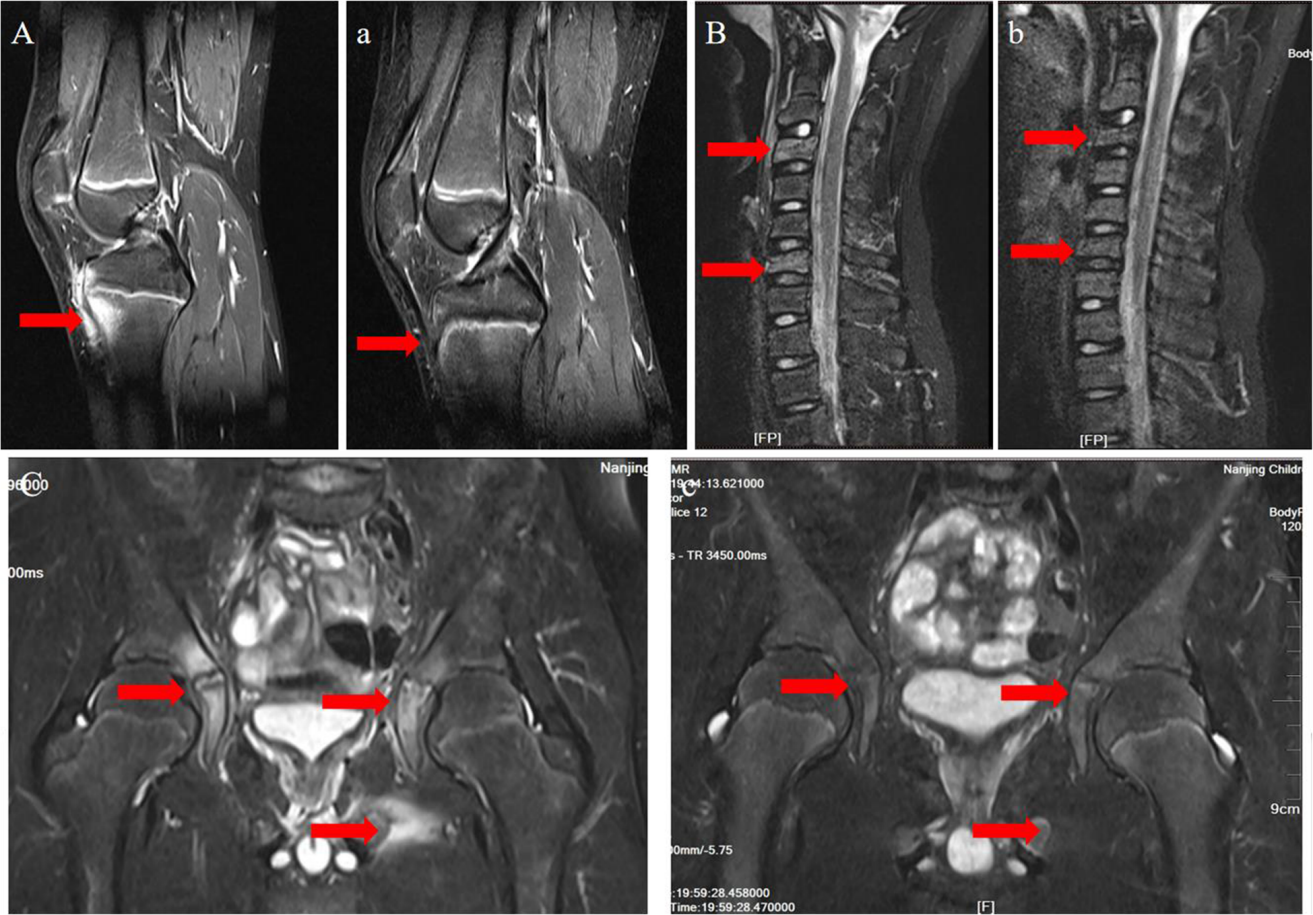
MRI findings in case 18 with chronic nonbacterial osteomyelitis MRI STIR or T2 show hyperintensity before treatment in tibia tubercle (**A**), cervicle vertebra (**B**) and hip joint (**C**), and remission after treatment (**a**, **b**, **c**)

**Fig. 2:**
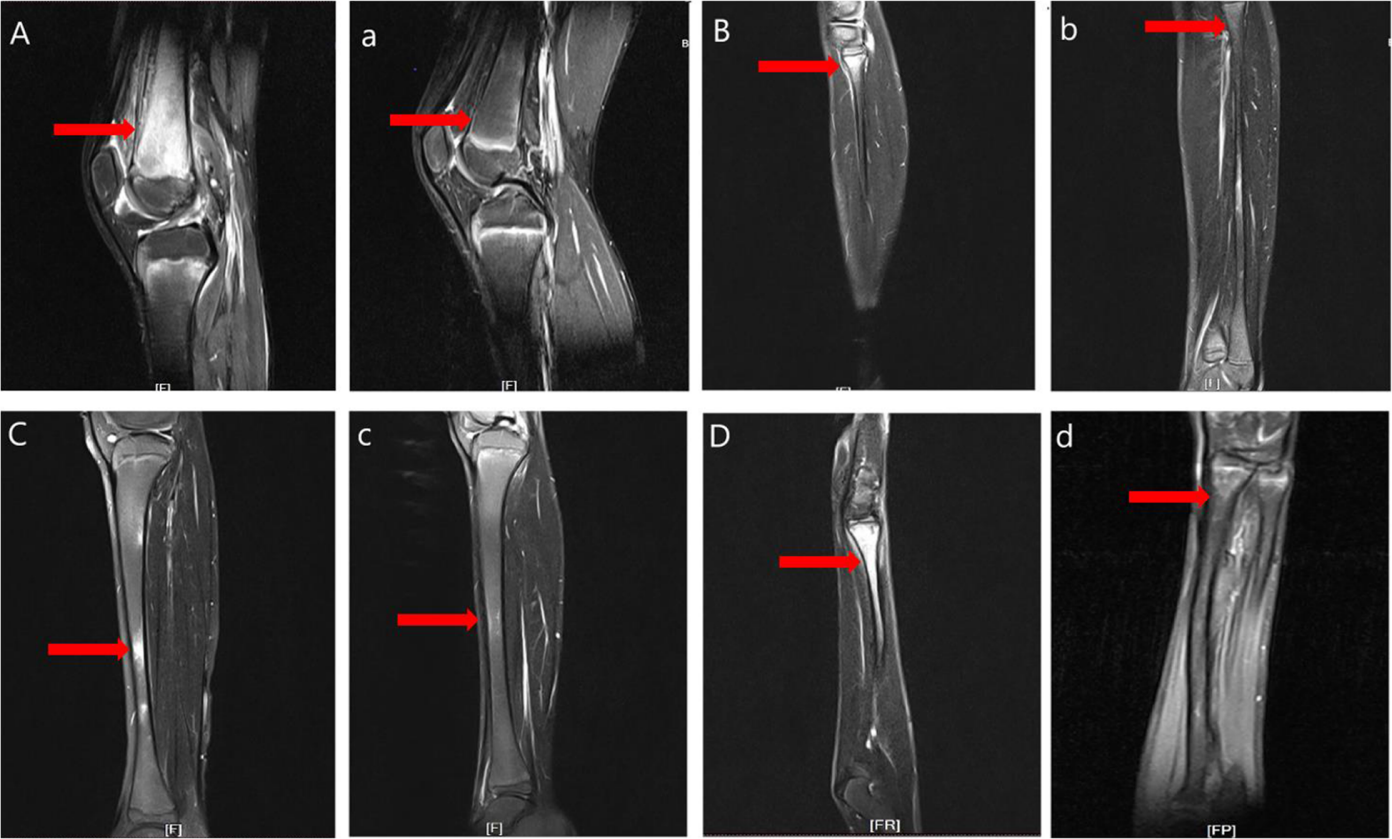
MRI findings in case 13 with chronic nonbacterial osteomyelitis. MRI STIR or T2 show hyperintensity of bone marrow before treatment in metaphysis or diaphysis of femur (**A**), fibula (**B**), tibia (**C**) and radius (**D**), and remission after treatment (**a**, **b**, **c**, **d**)

**Fig. 3:**
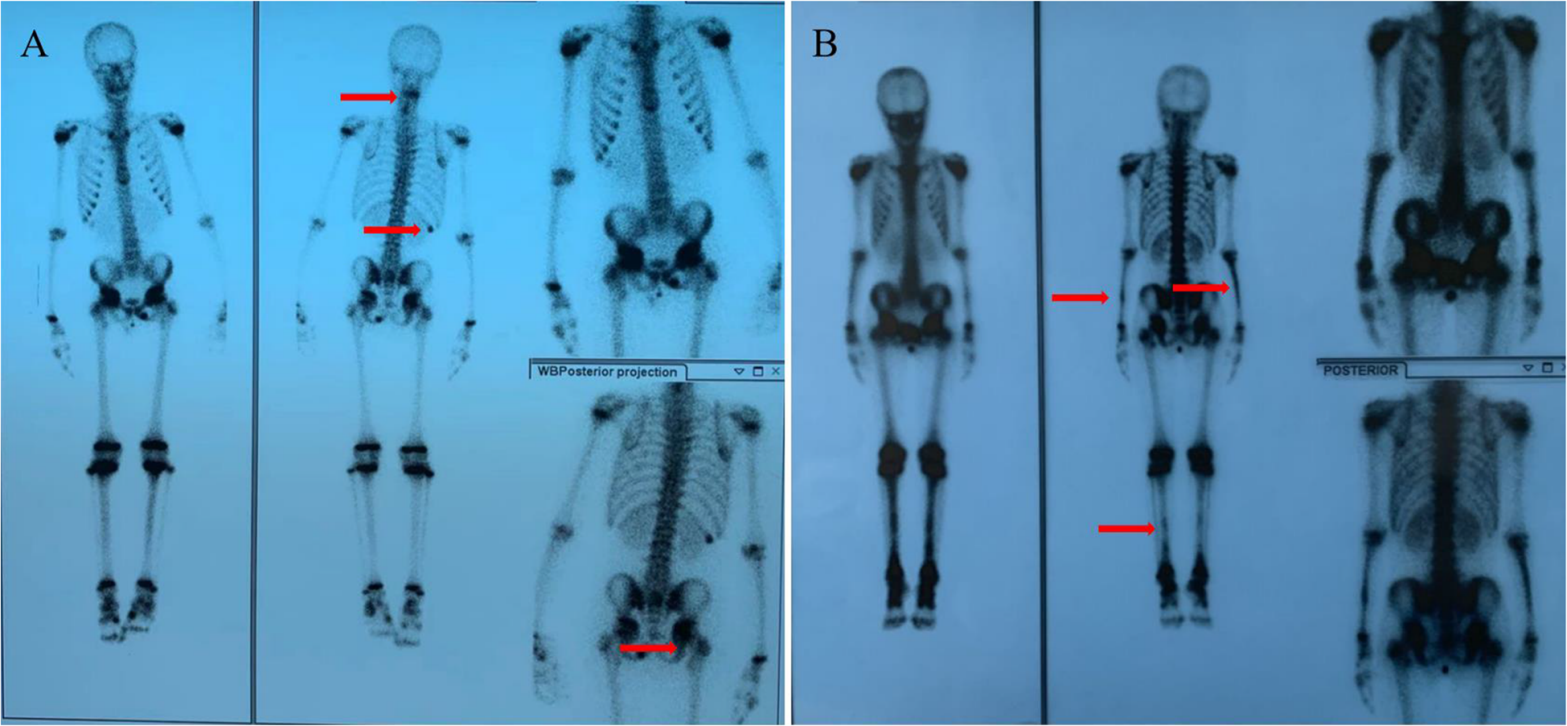
Bone scintigraphy findings in case 18 (**A**) and case 13 (**B**) with chronic nonbacterial osteomyelitis Bone scintigraphy showed increased tracer uptake in cervicle vertebra (**A**), the 12th rib (**A**), the right hip (**A**) and bilateral tibia fibula and ulna radius (**B**)

**Fig. 4:**
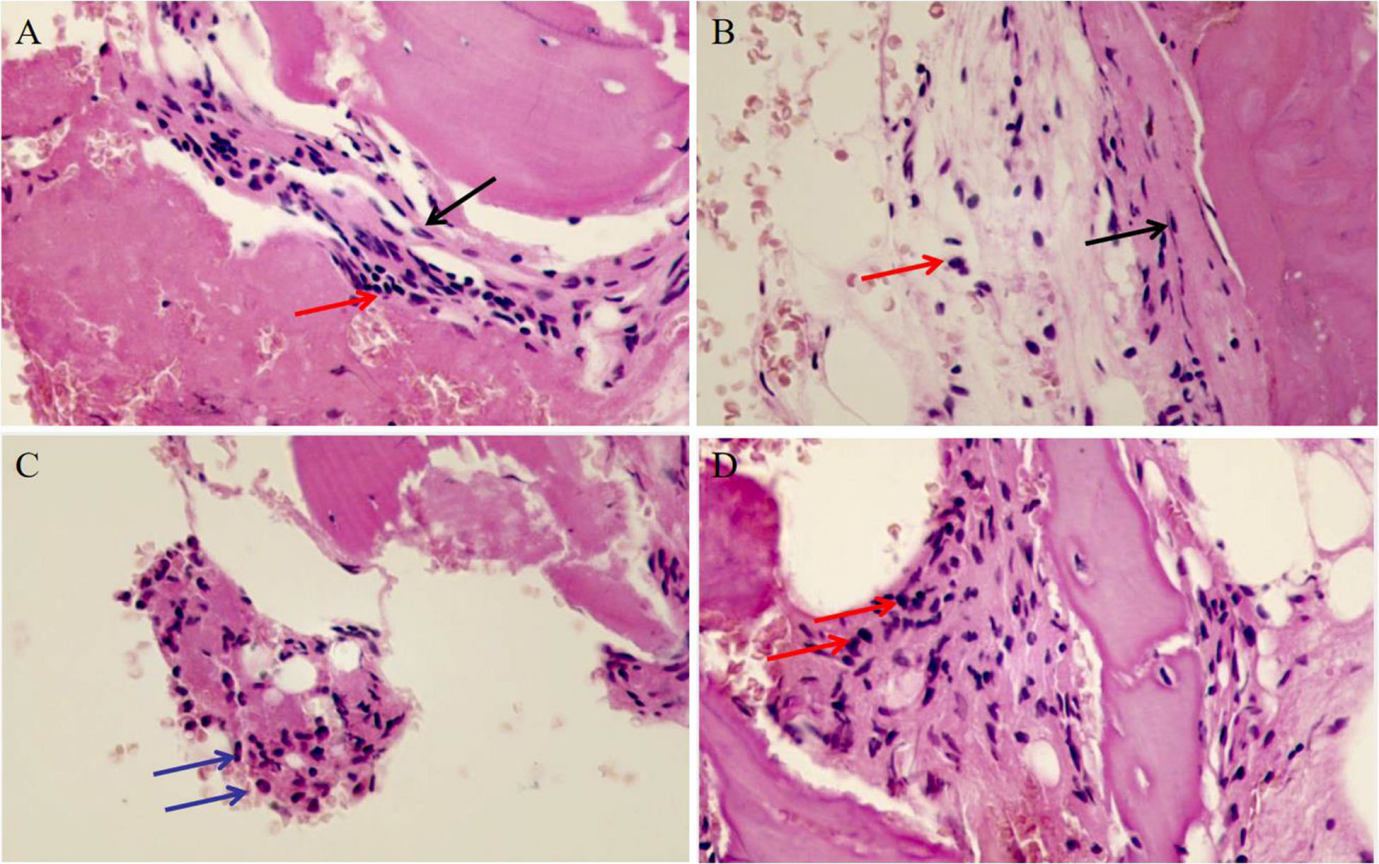
Pathological findings in patients with chronic nonbacterial osteomyelitis (400×). Adipose tissue and proliferating fibrous tissue (black arrow) were seen between bone trabeculae, scattered lymphocytes (red arrow) and plasma cells (blue arrow) were observed, without evidence of infection, malignancy, or histiocytosis

Results from imaging studies are detailed in Table 2. Among eight patients for whom plain radiographs were available for review, three showed Bony roughness, one showed bone enlargement and pathologic fracture and the others showed no abnormalities. Ten patients underwent computerized tomography, seven showed bone destruction, three showed uneven density of bone marrow, and one showed bony expansion. Seventeen patients underwent MRI of the main sites of localized pain, revealing abnormal bone findings in all cases: 82.3% showed increased signal in STIR, 11.7% bone edema, and 11.7% periostitis (Figs. 1 and 2 showed increased signal in STIR before and after treatment respectively). Twelve patients underwent three-phase Tc99 bone scintigraphy; in all of them, there was increased tracer uptake in the affected region (Fig. 3). Bone biopsy was performed in 11 patients (61.1%), showing adipose tissue and proliferating fibrous tissue and blood vessels were seen between bone trabeculae; scattered lymphocytes and plasma cells were observed, without evidence of infection, malignancy, or histiocytosis (Fig. 4).

**Table 2:** Imaging characteristics of patients with chronic nonbacterial osteomyelitis

	Number (frequency, %)
X-ray findings (*n* = 8)	
Bony roughness	3 (37.5%)
pathologic fracture	1 (12.5%)
CT findings (*n* = 10)	
bone destruction	7 (70%)
Uneven density of bone marrow	3 (30%)
bony expansion	1 (10%)
MRI findings (*n* = 17)	
bone edema	2 (11.7%)
periostitis	2 (11.7%)
Hyperintensity in bone marrow	14 (82.3%)
Hyperintensity in soft tissue	6 (35.3%)
Bone scintigraphy findings (*n* = 12)	
Increased uptake	12 (100%)

### Treatment

In terms of treatment, patients received NSAIDs (*n* = 16), methotrexate (*n* = 7), corticosteroids (*n* = 8), sulfasalazine (*n* = 2), bisphosphonates (*n* = 8), TNF-α inhibitor (*n* = 5), and thalidomide (*n* = 1). Decision of which therapeutic agent to use and in which order relied on the treating physician’s criteria. NSAIDs were used as the first-line therapy, followed by disease-modifying anti-rheumatic agents such as methotrexate or sulfasalazine. Patients who failed above treatments were placed on TNF inhibitors or bisphosphonates. The treatment protocols and outcomes of all patients were shown in Table 3. Three patients (case 3, case 13 and case 16) relapsed, two of whom (case3 and case 13) stopped taking drugs on their own. Case 15 and 16 received both TNF inhibitors and bisphosphonates therapy after NSAIDs, steroids and MTX therapy failed, which achieved good results. Three patients (case 1, case 12 and case 18) had spine involvement. Case 1 was treated with NSAIDs, SASP and steroids, achieved remission on medication. Case 12 was lost to follow up. Case 18 was treated with NSAIDs, MTX and pamidophosphate for 3 months with partial response. We had 11.1% remission without medicines in a median follow-up of 16 months.

**Table 3:** Treatment protocols of these 18 CNO patients

	NSAIDs	DMARDs	Steriod	TNFi	BPs	comments
Case 1	√	√	√			Treated with NSAIDs, SASP and steroids. Followed 18 months. Remission on medication.
Case 2						Treated with antibiotic for 2 weeks and completely responsed. Then the patient was followed 2 years and confirmed self-limited.
Case 3					√	Followed 6 months. The patient was treated with alendronate for 4 months, and achieved resonse. But relapsed after withdrawl by himself for 1 months. Then treated with alendronate again.
Case 4	√				√	Followed only 1 month. Remission on medications.
Case 5	√					Followed 5 months. Remission on medications.
Case 6	√	√	√	√		Treated with antibiotics and NSAIDs and steroids for 2 weeks with no response. Then added MTX and TNFi with full response. Followed 30 months, only treated with TNFi now.
Case 7	√		√		√	Treated with NSAIDs for 2 month with no response, then added steriods and alendronate with full response. Followed 10 months, with remission on medications.
Case 8	√	√	√			Treated with NSAIDs for 2 months with no response, then added steriods and MTX with full response. Followed 29 months, with remisssion on medications.
Case 9	√				√	Treated with NSAIDs for 3 years with partial response, then treated with alendronate with full resonse. Followed 4 years and 6 months, with remission on medications.
Case 10	√		√	√		Treated with NSAIDs for 2 weeks with no response, then added steriods and TNFi with full response. Followed 3 months with remission on medications.
Case 11	√	√	√			Treaed with NSAIDs, thalidomide and steriods with full response. Followed 2 years with complete remission.
Case 12	√		√			Lost to follow up.
Case 13	√	√		√		Treaed with NSAIDs and MTX and TNFi with full response. But relapsed after withdrawl by himself. Then treated with NSAIDs and MTX and TNFi again. Followed 11 months with remission on medications.
Case 14	√	√			√	Treated with NSAIDs for 6 months with partial response. Then added MTX and Pamidophosphate with partial response. Then treated with NSAIDs and MTX and Pamidophosphate and TNFi with full response. Followed 10 months with remission on medications.
Case 15	√	√		√	√	Treated with NSAIDs and MTX for 2 months with partial response, then added pamidophosphate and TNFi with full response. Followed 10 months with remission on medications.
Case 16	√	√	√	√	√	Treated with NSAIDs and pamidophosphate with full response. But relapse after 6 months, then added MTX and steriods with no response, then added TNFi with full response. Followed 2 years, with remission on medications.
Case 17	√	√				Followed only 2 months.
Case 18	√	√			√	Treated with NSAIDs, MTX and pamidophosphate for 3 months with partial response.

## Discussion

The present study was a multicenter series of CNO patients from five medical centers in South China. Compared with previous reports, the clinical characteristics of our patients were a little different. (details in Table 4) [8, 12, 15, 17–25] The female advantage in China is not such obvious as European countries [8, 12, 15, 18, 22–25]. The median age at diagnosis and diagnostic delays were 9.2 years and 10.2 months, respectively. The diagnostic delay ranged from 1 to 72 months, suggesting that CNO is still sometimes not well recognized in our country. In term of clinical manifestation, bone pain is still the most frequency symptom, but the fever rate in our study, which up to 44.4%, is more higher than that in other studies [17, 18, 20, 22, 24]. This may be related to bone inflammation. The most common bones involved in our study is the long bones of the limbs, especially the lower bones. In cases of Europe and USA, the clavicle, pelvis and spine are more frequently involved [8]. The comorbidities of our patients are mainly arthritis, only one patient has acne and gastrointestinal ulcer. The gastrointestinal symptoms, palmoplantar pustulosis, psoriasis and acne are more frequently in European cases [8, 12, 20]. It is also different from other studies, our CNO patients had no family history, no HLA-B27 positivity and no ANA positivity, suggesting a different genetic background.

**Table 4:** Comparison between our study and previous reports

	Patients, n	female, %	Age at disease on set, mean(y)	lesions	Initial syptoms	Comorbidities	treatment	CNO family history,%	Immunological indicators	Follow up, mean(m)
China, Ma L. et al., (present study)	18	56	9.2	94%:Mulifocal 6%:unifocal	100%:Pain 22%:swelling 28%:limp 44%:Fever	44.4%:Arthritis 5%:IBD 5%:skin lesions	1st line NSAIDS 2nd line: Steroids SSZ, MTX, Bisphosphonates and TNF blockers	0	0:HLA-B27 (+) 0:ANA (+)	16
United states, Gaal A, et al., (2020) [21]	22	36	11	18%:Multifocal 82%: Unifocal	ND	ND	1st line NSAIDS 2^nd^line: Steroids, DMARDS, Bisphosphonates and TNF blockers	ND	7%:HLA-B27(+) 30%:ANA (+)	ND
Chile, Concha S, et al. (2020) [19]	19	47	10	100%:Multifacal	ND	21%:Arthritis 0:IBD 0:skin lesions	1st line NSAIDS 2nd line: Steroids MTX, Bisphosphonates and TNF blockers (adalimumab)	ND	16%:HLA-B27(+) 37%:ANA (+)	ND
India, Rao A, et al. (2018) [17]	6	0	13	100%:Multifacal	100%:pain 33%:fever	ND	1st line: NSAID’S and Methotrexate 2nd line: Bisphosphonates/ TNF-blockers	ND	ND	31.5
Europe, Girschick H, et al.(2018) [8]	486	64	9.9	71%:Mulifocal 29%:unifocal	ND	29%:Arthritis 8%:IBD 14%:skin lesions	1st line NSAIDS 2nd line: Steroids SSZ, MTX, Bisphosphonates and TNF blockers	3	8%:HLA-B27 (+) 38%:ANA (+)	49
Germany Schnabel A, et al.,(2017) [20]	56	59	11	77%:Multifocal 23%: Unifocal	11%:fever	36%:Arthritis 11%:IBD 18%:skin lesions	1st line NSAIDS 2^nd^line: steroids, MTX, SSZ, Bisphosphonates, and TNF blockers	ND	21%:HLA-B27(+) 15%:ANA (+)	29
UK, Roderick MR, et al. (2016) [24]	41	76	9	76%:Multifocal 26%: Unifocal	15%:fever 17%:swelling	10%:skin lesions	1st line NSAIDS 2^nd^line: steroids, MTX, SSZ, Bisphosphonates, and TNF blockers	ND	ND	96
Germany, Silier CCG, et al. (2015) [18]	105	73	9.5	80%:Mulifocal 20%:unifocal	97%:Pain 60%:swelling 25%:redness 17%:Fever	9%:Arthritis 1%:IBD 19%:skin lesions	1st line NSAIDS and steroids 2^nd^line:Bisphosphonates and TNF blockers	15	ND	ND
France, Wipff J, et al. (2015) [12]	178	69	9.8	70%:Mulifocal 30%:unifocal	20%:fever	11%:Arthritis 33%:IBD 8%:skin lesions	1st line NSAIDS 2^nd^line: steroids, MTX, SSZ, Bisphosphonates, TNF blockers and anti–IL-1R (anakinra)	32	7%:HLA-B27 (+) 12%:ANA (+)	47
Australia, Walsh P, et al. (2015) [25]	34	62	9.8	82%:Multifocal 18%: Unifocal	ND	50%:Arthritis 3%:IBD, uveitis 36%:skin lesions	1st line NSAIDS 2nd line: Steroids MTX, AZA, Adalimumab	ND	9%:HLA-B27(+) 36%:ANA (+)	25
Germany, Beck C, et al. (2010) [23]	37	65	11	78%:Multifocal 22%: Unifocal	37%:swelling 22%:morning stiffness	38%:Arthritis 3%:IBD 17%:skin lesions	1st line Naproxen 2nd line: Sulfasalazine and steroids	ND	8%:HLA-B27(+) 59%:ANA (+)	6
France, Catalano-Pons C, et al., (2008) [22]	40	85	10	62%:Multifocal 38%: Unifocal	100%:pain 10%:swelling 23%:fever	10%:Arthritis 3%:skin lesions	1st line NSAIDS 2^nd^line: Steroids, SSZ, MTX, AZA Bisphosphonates and TNF blockers (etanercept)	ND	ND	ND
Germany, Jansson A, et al. (2007) [15]	89	65	10	81%:Multifocal 19%: Unifocal	ND	7%:IBD 25%:skin lesions	1st line NSAIDS 2^nd^line: Steroids, MTX, AZA, Bisphosphonates and TNF blockers (Infliximab)	12	0:HLA-B27(+) 33%:ANA (+)	ND

*TNF* Tumour necrosis factor, *NSAIDs* Non-steroidal anti-inflammatory drugs, *MTX* methotrexate, *SSZ* sulfasalazine, *AZA* azathioprine

On physical examination, swelling is infrequently observed, but when the disease is active, it is a sign of specific points of bone sensitivity [9]. CNO most frequently involves the long bones, followed by the pelvic bones, the vertebral column or the shoulder girdle/clavicle [3, 26]. The bones involved tend to be symmetrical, except the clavicle. Unifocal long bone involvement needs to be distinguished from culture-negative bacterial osteomyelitis by blood bacterial culture or bone marrow [27].

Laboratory tests of CRMO are not specific. Routine inflammatory parameters (WBC, white blood cell count; CRP, C reactive protein; ESR, erythrocyte sedimentation rate) are usually normal or mildly elevated. Imaging techniques are vital for diagnosing CNO and for excluding differential diagnoses [28]. Of our patients, 94.5% were evaluated with MRI, 52.9% with X-ray, and 58.5% with CT, while 64.7% had a bone scintigraphy. Compared with X-ray and CT, MRI is the most sensitive imaging technique to determine the extent and severity of bone involvement, particularly in the early stage. They can detect bone edema even before bone erosions and sclerosis develop and help assess the inflammation of surrounding tissues [29, 30]. More recently, whole-body MRI has been reported to be useful to screen the entire skeleton for bone lesions [31]. Bone scintigraphy is also useful for this purpose. It provides a global skeletal assessment at a lower cost [32], which can show abnormal concentrations of radionuclides, indicating the site of lesions, but cannot distinguish between inflammation and bone marrow metabolic hyperplasia. The radiation is also harmful to the body.

Bone biopsies are usually performed to exclude chronic infections, malignancies, or other systemic diseases, especially in patients with unifocal lesions [33]. In the present study, 47.1% of our patients underwent a bone biopsy. The histopathological findings of CNO are nonspecific inflammatory changes. A bone biopsy followed by pathological and pathogenic examination is very helpful for differential diagnosis.

To help diagnosis, we used the clinical NBO scores provided by Annette F. Jansson et al. [16] Although the score provides a reference standard for the diagnosis of the disease, CNO continues to be a diagnosis of exclusion. Important differential diagnoses include malignancies, infections, immunodeficiency, Langerhans cell histiocytosis (LCH), and other autoinflammatory disorders [33].

Although three consensus treatment plans (CTPs) were developed for CNO patients refractory to NSAID monotherapy by the Childhood Arthritis and Rheumatology Research Alliance (CARRA) [34], therapy protocols of CNO are not yet standardized. In general, first-line treatment is NSAIDs, which may reduce the pain and, in some cases, decrease the number of bone lesions in 3 months [35]. Second-line treatments usually includes methotrexate, corticosteroids, biologic drugs (mainly TNF-α inhibitors), and bisphosphonates depending on the severity of the disease and the presence of comorbidity and/or complications [36]. Almost all of our patients started treatment with NSAIDs but had to switch to other treatments because of partial response and relapse. Methotrexate was the second most frequent treatment, but six of these seven patients received steroids, bisphosphonates, or biologic drugs at the same time. Thus, it is difficult to assess the real impact of methotrexate. Different biologicals have been used to treat CNO, most commonly TNF-α inhibitors [25]. In our study, remission was achieved more frequently with TNF-α inhibitors, including adalimumab and etanercept. Bisphosphonates given to seven patients, resulted in remission in six patients. One patient experienced worsened bone pain after bisphosphonates, subsequently remitting on an adalimumab and bisphosphonate combination, which achieved a good result. Based on our clinical experiences, long bone lesions in diaphyses are more easily improved than those in epiphyses. It is worth noting that, spinal involvement can lead to fractures and secondary bone deformity [37]. This emphasizes the need for early diagnosis and aggressive treatment to prevent complications. Despite recent advances, there is no information on the optimal duration of treatment. Further studies about treatmnt are needed.

The long-term prognosis of CNO is generally favorable, with remission observed in 40% of patients after 1–5 years of follow-up [38]. In our study, we had a remission rate of 11.7% without medicines in a median follow-up of 16 months. The recurrence of the disease is very common. In a US cohort, a recurrence rate of 83% was observed after a follow-up of 1.8 years [21]. In our study, the recurrent rate was 17.6%. This may be related to the small sample size. It has been reported that patients can present a flare even 15 years after the onset of the disease, so it requires monitoring and long-term follow-up [38].

## Conclusion

This study is the first case series of CNO from South China to describe the features and outcomes of such an autoinflammatory bone disease. The diagnosis should include clinical history, laboratory and imaging examination, and histopathological examination. Other causes of chronic bone pain should be ruled out. For treatment, NSAIDs are used as first-line drugs followed by steroids, MTX, SASP, bisphosphonates, and TNF-α inhibitors. Combination therapy with bisphosphonates and TNF-α inhibitors may be an option for refractory CNO. The limitation of this study is its small sample size. Thus, further studies including more patients from other tertiary centers are required to formulate diagnostic and treatment strategies for CNO.

## Data Availability

The data and materials used in this study can be made available on request.

## Abbreviations

CNO: Chronic nonbacterial osteomyelitis
NSAIDs: Non-steroid anti-inflamatory drugs
MTX: Methotexate
SASP: Salazosulfadimidine
CRMO: Chronic recurrent multifocal osteomyelitis
SAPHO: Synovitis, acne, pustulosis, hyperostosis, and osteitis
CT: Computed tomography
ESR: Erythrocyte sedimentation rate
CRP: C-reactive protein
HLA: Human leukocyte antigen
ANA: Antinuclear antibodies
SSZ: Sulfasalazine
TNF: Tumor necrosis factor
